# A Decoupling Design with T-Shape Structure for the Aluminum Nitride Gyroscope

**DOI:** 10.3390/mi10040244

**Published:** 2019-04-12

**Authors:** Jian Yang, Chaowei Si, Guowei Han, Meng Zhang, Jin Ning, Yongmei Zhao, Fuhua Yang, Xiaodong Wang

**Affiliations:** 1 Engineering Research Center for Semiconductor Integrated Technology, Institute of Semiconductors, Chinese Academy of Sciences, Beijing 100083, China; yangjian@semi.ac.cn (J.Y.); hangw1984@semi.ac.cn (G.H.); zhangmeng@semi.ac.cn (M.Z.); ningjin@semi.ac.cn (J.N.); ymzhao@semi.ac.cn (Y.Z.); fhyang@semi.ac.cn (F.Y.); 2 College of Materials Science and Opto-Electronic Technology, University of Chinese Academy of Sciences, Beijing 100049, China; 3 State Key Laboratory of Transducer Technology, Chinese Academy of Sciences, Beijing 100083, China; 4 Center of Materials Science and Optoelectronics Engineering, University of Chinese Academy of Sciences, Beijing 100049, China; 5 School of microelectronics, University of Chinese Academy of Sciences, Beijing 100049, China; 6 Beijing Engineering Research Center of Semiconductor Micro-Nano Integrated Technology, Beijing 100083, China

**Keywords:** aluminum nitride, microelectromechanical systems (MEMS), gyroscope, piezoelectric effect, orthogonal coupling

## Abstract

This paper reports a novel design for the decoupling of microelectromechanical systems (MEMS) gyroscopes. The MEMS gyroscope is based on piezoelectric aluminum nitride (AlN) film, and the main structure is a mass hung by T-shape beams. A pair of parallel drive electrodes are symmetrically placed on the surface of the vertical bar for driving the oscillating mass. A serpentine sense electrode is placed on the lateral bar. When the gyroscope is oscillating in drive mode, charges with equal quantity and opposite sign will be polarized and distributed symmetrically along the lateral bar. These charges neutralize each other at the sense electrode. Therefore, no coupling signals can be detected from the sense electrode. This design can realize the decoupling between the drive mode and sense mode. In this work, the T-shape decoupled structure was designed as the key component of an AlN piezoelectric gyroscope and the whole structure was simulated by COMSOL Multiphysics 5.2a. The working principle of the decoupling is described in detail. Electrical properties were characterized by the dynamic signal analyzer. According to the test results, the drive mode and the sense mode are decoupled. The coefficient of orthogonal coupling is 1.55%.

## 1. Introduction

Microelectromechanical systems (MEMS) gyroscopes play an important role in inertial navigation systems. They are widely used in automatic drive, industrial control, unmanned aerial vehicle and consumer electronics [1,2,3,4]. The working principles of these gyroscopes are based on the Coriolis’ effect [5]. In the past decade, the performance of MEMS gyroscopes has improved continuously, and approached the requirements of tactical-grade and navigation-grade applications [6,7,8]. However, there are some factors limiting the performance of MEMS gyroscopes. Orthogonal coupling is one of the main restrictions for the sensitivity and bias instability of MEMS gyroscopes [9,10,11]. Owing to the manufacturing tolerances or design of the structure, the mechanical crosstalk of the drive mode couples to the sense electrodes. This reduces the performance of MEMS gyroscopes, for example, the scale factor linearity, bias instability and operating range.

To overcome the orthogonal coupling, many approaches have been taken. These approaches include the use of electrostatic tuning [9,12,13,14], the drive electrode with one degree of freedom (1-DOF) motion [15], or the sense electrode with 1-DOF [16,17]. Generally, an elaborate mechanical structure should be designed to restrict the movement of the structure in the orthogonal direction. However, it is very difficult to realize decoupling and the effect is limited. In the reported work of Alper and Akin [16], the mechanical coupling from the drive mode to the sense mode is 2% of the drive mode vibration amplitude. There is always a physical connection between the drive mode oscillator and the sense frame, which is in order to couple the rotation-induced Coriolis forces acting on the oscillating mass to the sensing frame. 

The piezoelectric gyroscope is the main type of MEMS gyroscopes, such as quartz, PZT (lead zirconate titanate) and aluminum nitride (AlN) ones [18,19,20]. The electrodes are placed on the surface of piezoelectric film [18,21]. Based on the piezoelectric effect, a mechanical deformation will polarize electric charges. The neutralization of coupling-induced charges is a new method for decoupling. 

T-shape beams have been thoroughly researched as MEMS resonators [22,23]. In this paper, we report a decoupled design, which is based on the piezoelectric properties of AlN film. A T-shape beam is proposed as a decoupled structure for the AlN MEMS gyroscope. A pair of parallel electrodes and a serpentine electrode were designed, based on the FEM (Finite Element Method) eigenmode simulation of displacement and stress. The mechanical crosstalk will lead to a deformation of the sense beam. Then, equal quantity of positive charges and negative charges will be polarized on the sense electrode simultaneously. These coupling-induced charges will neutralize each other and no signal will output from the sense electrode. Therefore, although there is a mechanical coupling between the drive mode structure and sense electrode, the charge neutralization can realize the decoupling between the drive mode signal and sense mode signal.

## 2. Design

AlN is a kind of piezoelectric material, and the converse piezoelectric coefficient *d*_31_ is −2.6 pm/V. Based on the piezoelectric effect, an AlN beam can be excited to bend in-plane by a couple of parallel electrodes. The working principle is described in [24].

**Figure 1:**
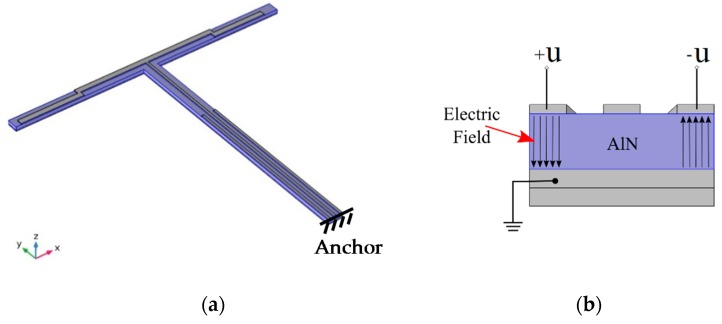
The T-shape structure (**a**) and the cross-section of the beam (**b**).

In this paper, a cross beam structure (T-shape) was designed, as shown in Figure 1. A couple of parallel electrodes were placed on the two sides of the vertical beam, which is along the *y* axis. They work as drive electrodes. Inverse voltages (±*u*) were placed on the two drive electrodes, respectively. The voltages can excite a couple of inverse stresses (±*σ*) and drive the whole structure to bend in-plane. Because of the Coriolis’ effect, a z axial angular rate can be detected by the lateral beam, which is along the *x* axis. 

The formula of *σ* is shown as Equation (1), which is deduced on the converse piezoelectric effect.

(1) $$\begin{array}{c} \sigma =E_{\mathrm{AlN}}\frac{u}{d_{\mathrm{AlN}}}d_{31}=\frac{E_{\mathrm{AlN}}u_{0}\mathrm{Sin}(\omega t)d_{31}}{d_{\mathrm{AlN}}}\\ u=u_{0}\mathrm{Sin}(\omega t) \end{array}$$

where *E*_AlN_ is the Young’s modulus of AlN, *d*_AlN_ is the thickness of AlN film, *u*_0_ is the amplitude of the drive voltage, *ω* is the angular frequency of drive voltage, and *d*_31_ is the converse piezoelectric coefficient of AlN, *d*_31_ = −2.6 pm/V.

**Figure 2:**
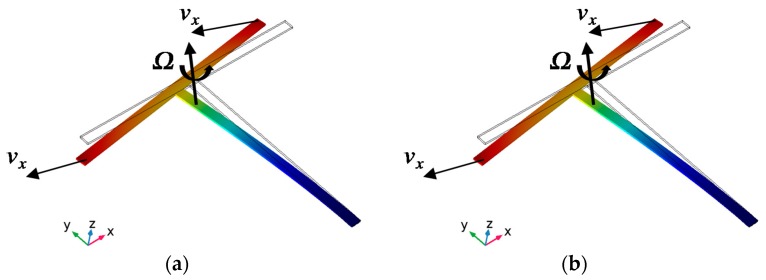
The bending vibration of T-shape structure. Drive mode (**a**) and sense mode (**b**).

The couple stresses will form a bending moment *M* and drive the T-shape structure to bend in-plane, as shown in Figure 2a. The formula of *M* is shown as Equation (2). This vibration can be designed as drive mode. Because of the Coriolis’ effect, Figure 2b will be the sense mode for the detection of z axial angular rate.

(2) $$\begin{array}{cc} M & =\sigma W_{\mathrm{dri}}d_{\mathrm{AlN}}(W-W_{\mathrm{dri}})\\ & =E_{\mathrm{AlN}}U_{0}\mathrm{Sin}(\omega t)d_{31}W_{\mathrm{dri}}(W-W_{\mathrm{dri}}) \end{array}$$

**Figure 3:**
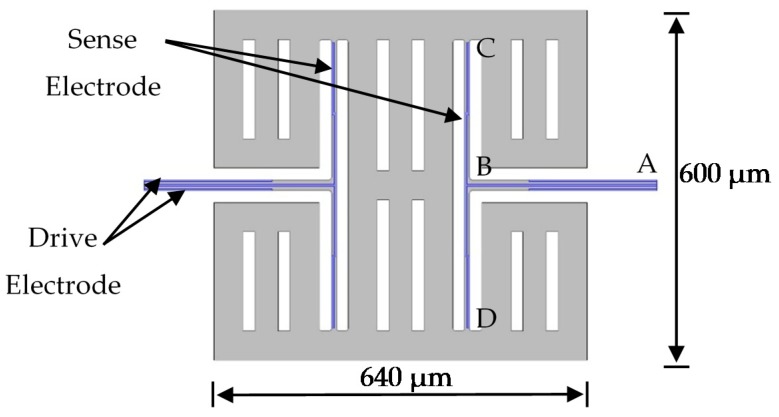
The schematic of aluminum nitride (AlN) microelectromechanical systems (MEMS) gyroscope.

**Figure 4:**
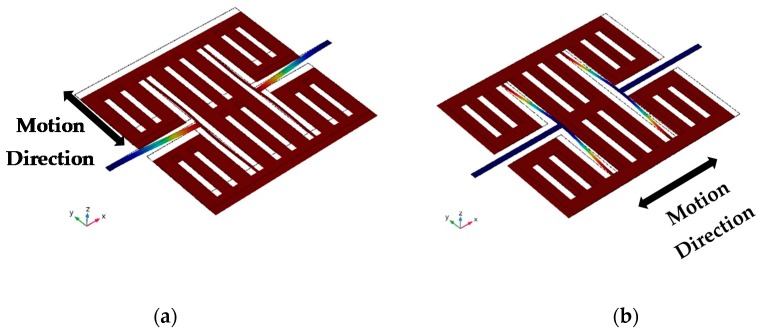
The displacement simulation of the drive mode (**a**) and sense mode (**b**) of an AlN MEMS gyroscope.

Based on the T-shape component, the structure of the AlN gyroscope was designed as shown in Figure 3. This gyroscope contains two T-shape structures and one mass. The symmetric structure can provide differential signals for drive mode and sense mode. The drive electrodes and sense electrodes are as shown in Figure 3. We simulated and optimized this gyroscope by COMSOL Multiphysics 5.2a. The dimensions of this MEMS gyroscope are shown in Table 1. The eigenmodes of this MEMS gyroscope were simulated by COMSOL Multiphysics. The drive mode and sense mode are as shown in Figure 4. Both of the two modes are in-plane vibration.

**Figure 5:**
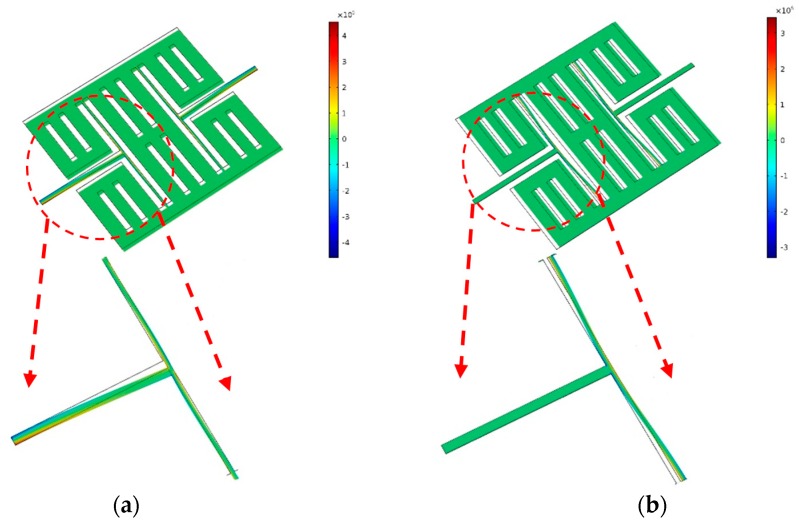
The stress simulation of the drive mode (**a**) and sense mode (**b**) of AlN MEMS gyroscope.

The piezoelectric charges are polarized by the mechanical stresses. To design the electrodes, the stress distribution should be simulated. Figure 5 shows the stress simulation of the drive mode and sense mode, respectively. From the simulation, we can get that the stresses are mostly located at the T-shape structure.

**Figure 6:**
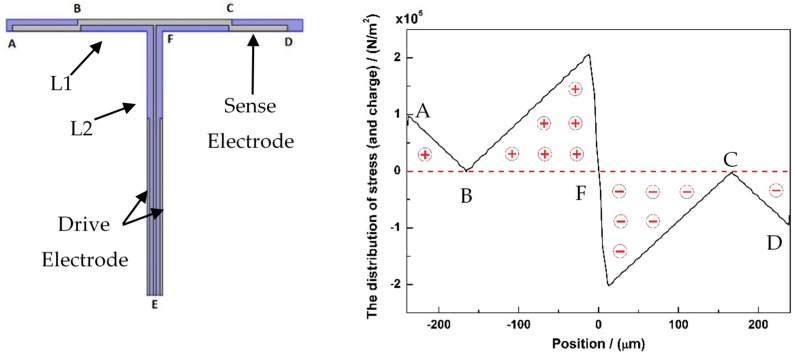
The stress distribution of drive mode along the sense electrode—ABCD. The positive/negative stresses will polarize positive/negative charges on the interface of AlN and electrodes.

The sense electrode was designed as shown in Figure 6, which contains two parts, EF and ABCD. The ABCD part is a serpentine electrode. All the sense electrodes are symmetric about the center line of T-shape. Based on the stress simulation of drive mode, as shown in Figure 5a, the points B and C are placed at the position of zero stress. Therefore, the stresses emerge anti-symmetrically along the serpentine electrode ABCD, about the center point F. Figure 6 shows the stress simulation results. The positive stresses and the negative stresses are anti-symmetric. According to the direct piezoelectric effect, the polarization charges are proportional to the force (or stress), as shown in Equation (3).

(3) $$Q=D_{\mathrm{AlN}}\cdot XD_{\mathrm{AlN}}=\left[\begin{array}{cccccc} 0 & 0 & 0 & 0 & 4 & 0\\ 0 & 0 & 0 & 4 & 0 & 0\\ -2 & -2 & 5 & 0 & 0 & 0 \end{array}\right]pC/N$$

where *Q* is the polarization charges, *D*_AlN_ is the direct piezoelectric coefficient (*pC*/*N*) of AlN, and *X* is the force.

As a result, the positive charges and the negative charges, which are produced by the piezoelectric effect will neutralize each other on the serpentine electrode ABCD. The EF electrode is placed at the center line of the T-shape beam. The charges will also neutralize each other on the electrode EF. Therefore, there will be no charge on the whole sense electrode at the drive mode. This means no orthogonal coupling at drive mode.

For the sense mode, there is no deformation of the L2 beam of the T-shape, as shown in Figure 4b. The stress is zero at the positions of the drive electrodes, as shown in Figure 5b. Therefore, no charge can be detected from the drive electrodes at the sense mode. This means no orthogonal coupling at sense mode.

**Figure 7:**
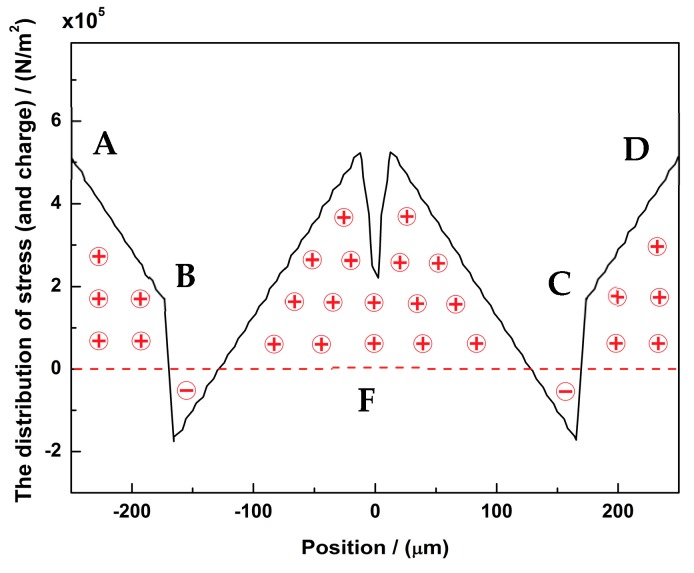
The stress distribution of sense mode along the sense electrode—ABCD. The positive/negative stresses will polarize positive/negative charges on the interface of AlN and electrodes.

At the sense mode, there will be a deformation at the L1 beam. Therefore, polarization charges will emerge. The distribution of stresses and charges along the ABCD electrode is shown in Figure 7. Few anti-charges will be neutralized on the electrode. Most of charges will survive and can be detected from the sense electrode.

## 3. Fabrication Process

**Figure 8:**
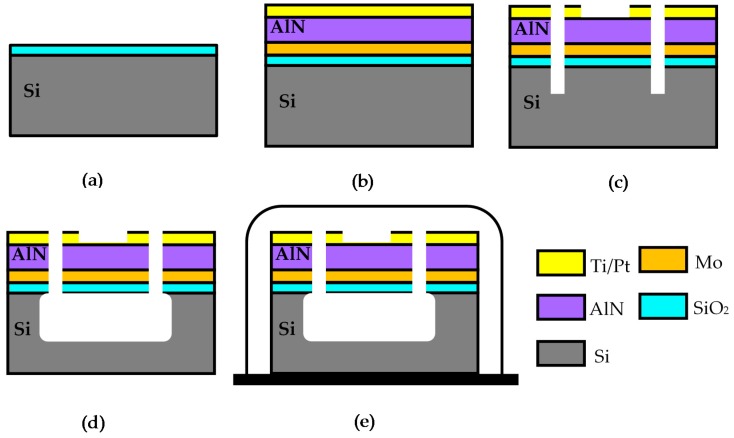
The fabrication process flow of the gyroscope: (**a**) The SiO_2_ layer is grown by the thermal oxidation process. The thickness of SiO_2_ layer is 300 nm; (**b**) The Mo layer works as the bottom electrode and is sputtered on the SiO_2_ layer. The (002) oriented AlN film is sputter on the Mo layer by magnetron sputtering process. The full width at half maximum (FWHM) of AlN film is 1.288° tested by X-ray diffraction (XRD). The Ti/Pt layer works as the top electrode, sputtered on the AlN film; (**c**) Different ICP etching processes are performed on the Ti/Pt, AlN, Mo, SiO_2_ and Si successively; (**d**) The releasing process is performed by inductively coupled plasma (ICP) isotropic etching process of Si. The etching gas is SF_6_; (**e**) The vacuum packaging of chip-level is used for sealing the gyroscope.

This prototype structure was fabricated on the n-type (100) Si substrate. The (002) oriented AlN film was deposited by magnetron sputtering process [25,26,27]. The thickness of AlN is 1.3 μm. The bottom electrode layer is Mo and is 300 nm thick. The top electrode layer is Pt/Ti with a thickness of 150 nm. In the fabrication, the patterning processes are the key points. The AlN film was etched by inductively coupled plasma (ICP) etching process with Cl_2_/BCl_3_/Ar [28]. The whole structure was released from Si substrate by ICP isotropic etching process with SF_6_. The process flow is shown in Figure 8 and is described as follows.

**Figure 9:**
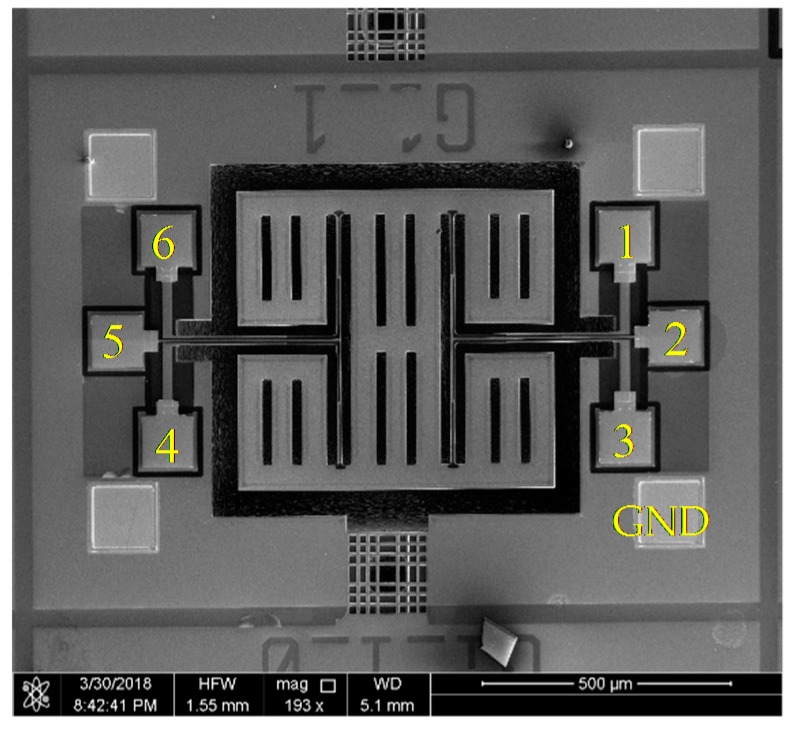
Scanning electron microscope (SEM) view of the AlN MEMS gyroscope.

**Figure 10:**
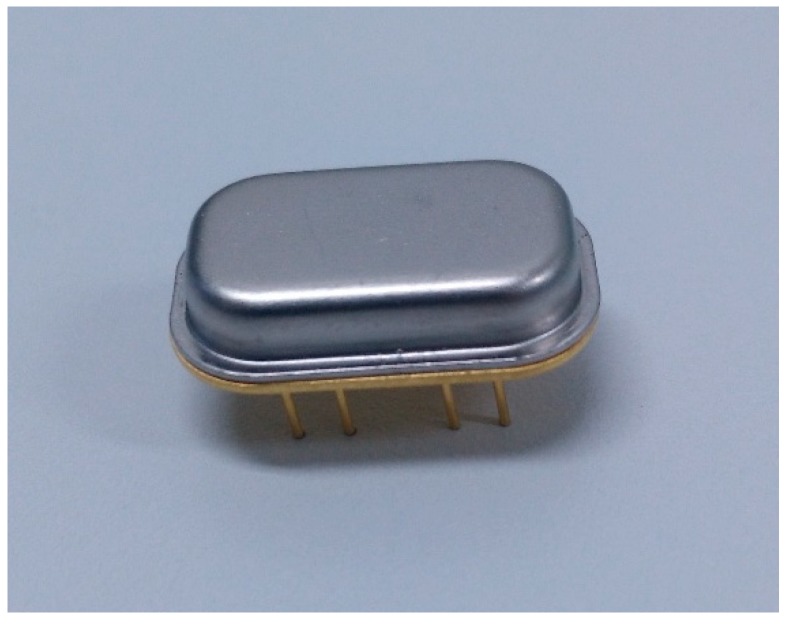
Metal vacuum packaging for the AlN MEMS gyroscope.

Figure 9 is the scanning electron microscope (SEM) picture of the fabricated gyroscope. The whole structure is symmetrical. 1,3,4,6 are symmetrical drive electrode pads. 2 and 5 are symmetrical sense electrode pads. A vacuum packaging process of chip-level was used. This offers the vacuum environment, high reliability and long-term stability for the MEMS gyroscope. In this work, the pressure of the vacuum packaging is 0.16 Torr. Figure 10 shows the metal vacuum packaging for the gyroscope.

## 4. Results and Discussion

**Figure 11:**
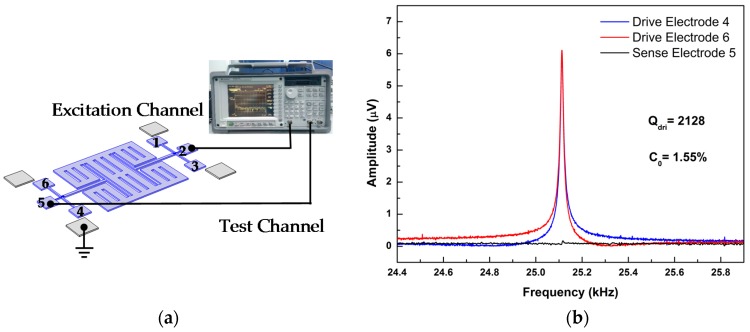
The test schematic (**a**). The frequency domain response of drive mode (**b**). Excited by the drive electrode 1 (or 3).

**Figure 12:**
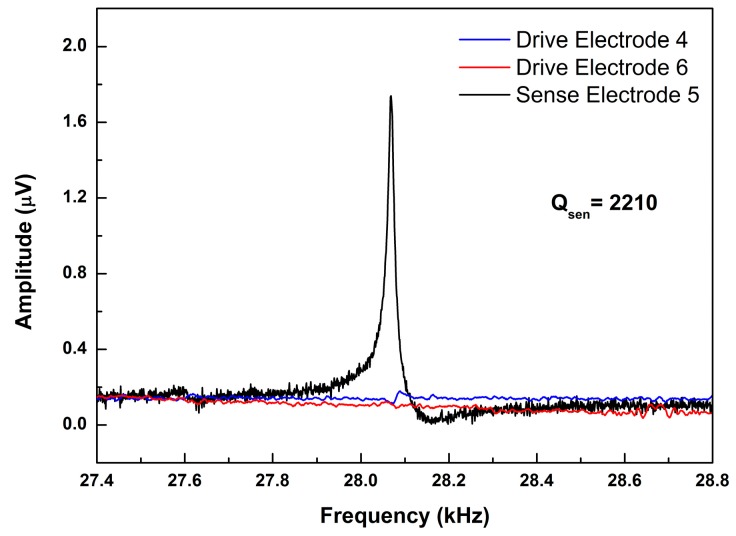
The frequency domain response of sense mode. Excited by the sense electrode 2.

The prototype device was tested by the dynamic signal analyzer Agilent 35670A (Santa Clara, CA, USA) as shown in Figure 11a. Figure 11b and Figure 12 show the electrical characterization of the drive mode and sense mode, respectively. The resonant frequency of the drive mode is 25.11 kHz, and the sense mode is 28.07 kHz.

For the drive mode, the excitation signal was applied on the drive electrode 1 (or 3) to excite the gyroscope. The opposite electrodes (4, 5 and 6) were connected to the test channel of the dynamic signal analyzer. The results are shown as Figure 11b. The obvious resonant peaks, which come from the drive electrodes 4 and 6, have equal amplitude and opposite phase. The quality factor of drive mode *Q*_dri_ is 2128. However, there is a weaker resonant peak on sense electrode 5 at 25.11 kHz. This is the coupling signal from the drive mode. The coefficient of orthogonal coupling *C*_0_ is 1.55%. The *C*_0_ is defined as Equation (4).

(4) $$C_{0}=\frac{A_{\mathrm{sen}}}{A_{\mathrm{dri}}}\times 100\%$$

where *A*_sen_ is the signal amplitude from the sense electrode, and *A*_dri_ is the signal amplitude from the drive electrode [29,30].

The coupling signal, which is detected from the sense electrode, comes from the process error, such as the size error of beams and electrodes, the asymmetry of serpentine electrodes and the non-homogeneity of material. These test results coincide with the theoretical analysis. It proves that this gyroscope with T-shape structures is decoupled at the drive mode.

For the sense mode, the excitation signal was applied on the sense electrode 2 to excite the sense mode. The opposite electrodes (4, 5 and 6) were tested by the dynamic signal analyzer. An obvious resonance peak, 28.07 kHz, appeared at the opposite sense electrode 5. The quality factor *Q*_sen_ is 2210. This means the sense electrode with a serpentine shape can detect the sense mode signal effectively. Most of the polarized charges survived at the sense electrode. However, there were extremely weak resonant peaks at the drive electrode 4 and 6 as shown in Figure 12. The two weak peaks come from the process error. These test results prove that this gyroscope with T-shape structures is decoupled at the sense mode.

## 5. Conclusions

This paper introduces a piezoelectric decoupled method. A T-shape structure, which is a key component is proposed and analyzed in detail. Based on the T-shape structure, a symmetric gyroscope structure was designed and optimized. The cross-coupling characteristic was analyzed. Based on the piezoelectric effect and the design of T-shape structure, the coupling polarized charges neutralize each other. No coupling signals will output from the sense electrode. Therefore, this MEMS gyroscope has a decoupled characteristic. In this work, the AlN gyroscope was fabricated by the MEMS process. A frequency domain test was performed for this fabricated. The quality factors of this AlN gyroscope are *Q*_dri_ = 2128, and *Q*_sen_ = 2210. The test results show that the coefficient of orthogonal coupling is 1.55%. The quadrature error comes from the process error, such as the size error of beams and electrodes, and the asymmetry of serpentine electrodes. In the future, we will focus on the improvement of fabrication processes and the signal processing to decrease the orthogonal coupling further.

## Figures and Tables

**Table 1:** The parameters of the MEMS gyroscope.

Parameters	Dimensions
Length of T-shape beam (AB)	322 μm
Width of T-shape beam (AB)	18 μm
Length of T-shape beam (CD)	500 μm
Width of T-shape beam (CD)	8 μm
Length of drive electrode	220 μm
Width of drive electrode	4.5 μm
Width of sense electrode	4 μm
Length of the proof mass	640 μm
Width of the proof mass	600 μm
